# Cerium Dioxide Nanoparticle Exposure Improves Microvascular Dysfunction and Reduces Oxidative Stress in Spontaneously Hypertensive Rats

**DOI:** 10.3389/fphys.2015.00339

**Published:** 2015-11-17

**Authors:** Valerie C. Minarchick, Phoebe A. Stapleton, Edward M. Sabolsky, Timothy R. Nurkiewicz

**Affiliations:** ^1^Center for Cardiovascular and Respiratory Sciences, West Virginia University School of MedicineMorgantown, WV, USA; ^2^Department of Physiology and Pharmacology, West Virginia University School of MedicineMorgantown, WV, USA; ^3^Department of Mechanical and Aerospace Engineering, West Virginia UniversityMorgantown, WV, USA

**Keywords:** microcirculation, cerium dioxide nanoparticles, hypertension, reactive oxygen species, anti-oxidant

## Abstract

The elevated production of reactive oxygen species (ROS) in the vascular wall is associated with cardiovascular diseases such as hypertension. This increase in oxidative stress contributes to various mechanisms of vascular dysfunction, such as decreased nitric oxide bioavailability. Therefore, anti-oxidants are being researched to decrease the high levels of ROS, which could improve the microvascular dysfunction associated with various cardiovascular diseases. From a therapeutic perspective, cerium dioxide nanoparticles (CeO_2_ NP) hold great anti-oxidant potential, but their *in vivo* activity is unclear. Due to this potential anti-oxidant action, we hypothesize that injected CeO_2_ NP would decrease microvascular dysfunction and oxidative stress associated with hypertension. In order to simulate a therapeutic application, spontaneously hypertensive (SH) and Wistar-Kyoto (WKY) rats were intravenously injected with either saline or CeO_2_ NP (100 μg suspended in saline). Twenty-four hours post-exposure mesenteric arteriolar reactivity was assessed via intravital microscopy. Endothelium-dependent and –independent function was assessed via acetylcholine and sodium nitroprusside. Microvascular oxidative stress was analyzed using fluorescent staining in isolated mesenteric arterioles. Finally, systemic inflammation was examined using a multiplex analysis and venular leukocyte flux was counted. Endothelium-dependent dilation was significantly decreased in the SH rats (29.68 ± 3.28%, maximal response) and this microvascular dysfunction was significantly improved following CeO_2_ NP exposure (43.76 ± 4.33%, maximal response). There was also an increase in oxidative stress in the SH rats, which was abolished following CeO_2_ NP treatment. These results provided evidence that CeO_2_ NP act as an anti-oxidant *in vivo*. There were also changes in the inflammatory profile in the WKY and SH rats. In WKY rats, IL-10 and TNF-α were increased following CeO_2_ NP treatment. Finally, leukocyte flux was increased in the SH rats (34 ± 4 vs. 17 ± 3 cells/min in the normotensive controls), but this activation was decreased following exposure (15 ± 2 vs. 34 ± 4 cells/min). These results indicated that CeO_2_ NP may alter the inflammatory response in both SH and WKY rats. Taken together, these results provide evidence that CeO_2_ NP act as an anti-oxidant *in vivo* and may improve microvascular reactivity in a model of hypertension.

## Introduction

There is a growing interest in developing therapeutic interventions that result in more precise and individualized treatment. Engineered nanomaterials (ENM) are prime candidates for therapeutic applications for several reasons (Kelkar and Reineke, 2011). First, the size of ENM (≤ 100 nm in at least one dimension) creates a large surface area for the attachment of various pharmaceuticals and/or imaging agents (Borm et al., 2006). Their small size also allows ENM to access every bodily component via the circulation (Stapleton and Nurkiewicz, 2014). Secondly, by altering the surface chemistry and/or using specific ENM, they may be precisely aimed at target areas in the body, which (in conjunction with a high surface) reduces the dosage needed to treat certain pathologies (Roco et al., 2010; Yu et al., 2010a). Finally, many ENM possess inherent unique characteristics (e.g., auto-fluorescence, and anti-oxidant activity) that can be exploited to increase their pharmaceutical relevance (Borm et al., 2006).

Cerium dioxide nanoparticles (CeO_2_ NP) are one type of ENM that are currently being investigated for their possible therapeutic significance (Colon et al., 2009; Kim et al., 2012; Madero-Visbal et al., 2012). CeO_2_ NP have catalytic activity, which arises from the presence of two valence states (Ce^3+^ and Ce^4+^) (Celardo et al., 2011). Due to the oxygen vacancies present in these nanoparticles, they are able to react with surrounding reactive oxygen species (ROS), thus introducing CeO_2_ NP as a potential *in vivo* mimetic for endogenous anti-oxidants like superoxide dismutase (Heckert et al., 2008; Xu and Qu, 2014; Dunnick et al., 2015).

Like many pharmaceuticals, CeO_2_ NP research has produced conflicting results about the efficiency of its potential anti-oxidant activity. *In vitro* analysis has shown an increase in ROS generation and inflammation following CeO_2_ NP co-incubation; however, other studies with cardiac progenitor and endothelial cells have shown a decrease in both these parameters (Park et al., 2008; Gojova et al., 2009; Horie et al., 2011; Wingard et al., 2011; Pagliari et al., 2012). The *in vivo* analysis of these nanoparticles is even more complex. Experimentally, injected CeO_2_ NP have been shown to decrease tissue damage, which is commonly associated with radiation treatments and strokes (Colon et al., 2009; Kim et al., 2012). Despite these promising results, CeO_2_ NP injected into young healthy rats may have detrimental effects that result in microvascular dysfunction (Minarchick et al., 2015). Currently, it is unclear if the potential positive effects of CeO_2_ NP are pathology specific. Furthermore, it is also unknown how CeO_2_ NP specific alterations in ROS production affect microvascular function.

Hypertension affects one out of three Americans and is a leading factor in the development of cardiovascular disease (Nwankwo et al., 2013). This pathology is associated with microvascular dysfunction that is, at least partially, attributed to increased ROS generation due to chronic inflammation, mitochondrial dysfunction, and/or nitric oxide synthase (NOS) uncoupling (Félétou and Vanhoutte, 2006; Brito et al., 2015). In addition to decreasing vasoactive factors directly, the elevated levels of ROS are capable of increasing steroid hormone production, which can impair NOS activity and prostaglandin formation, which increase the risk of organ damage (Suzuki et al., 1995). The influence of ROS on microvascular function has led to the investigation of anti-oxidants as potential therapeutic treatments (Brito et al., 2015). If the anti-oxidant potential of CeO_2_ NP were better understood, this ENM could be developed as a treatment to decrease high levels of ROS, which may ultimately reduce the microvascular dysfunction associated with various diseases such as hypertension.

Therefore, the purpose of this study was to assess the *in vivo* anti-oxidant potential of CeO_2_ NP in a high ROS environment, specifically hypertension. Previous *in vitro* studies have shown that in the presence of elevated ROS CeO_2_ NP act as an anti-oxidant and can decrease ROS (Minarchick et al., 2015). Based on these results, we hypothesize that following injection CeO_2_ NP will act as an anti-oxidant and reduce the oxidative stress and microvascular dysfunction that is associated with hypertension. This potential outcome may provide valuable information about the anti-oxidant activity of CeO_2_ NP, which is pivotal for it to be developed as an anti-oxidant treatment for cardiovascular diseases.

## Methods and materials

### Cerium dioxide nanoparticle (CeO_2_ NP) production and characterization

CeO_2_ NP powders were synthesized by a hydrothermal process as previously described (Minarchick et al., 2013). Briefly, cerium (IV) ammonium nitrate (99+% Alfa Aesar, Ward Hill, MA) was added to de-ionized water (H_2_O) and this solution was added drop-wise into a basic solution of tetramethylammonium hydroxide pentahydrate and de-ionized H_2_O. The pH of the dispersion was altered to ~10.5 with ammonium hydroxide and was maintained throughout the reaction. The dispersion was placed in a 300 ml Autoclave Engineers EZE-Seal autoclave (Erie, PA) at 240°C for 1 h. Once removed from the autoclave, the dispersion was placed into a centrifuge and the liquid was removed and replaced with ethanol. After the washing step, the dispersion was dried at 60°C overnight and sieved through a 200 mesh screen for characterization. The nanoparticles were previous characterized (Minarchick et al., 2013). Briefly, the CeO_2_ NP had a surface area of 81.36 m^2^/g measured by Micromeritics ASAP 2020 (Norocross, GA). JEOL JEM-2100 High Resolution Transmission Electron Microscope (TEM) (Peabody, MA) determined the primary particle size (~2–4 nm) and shape (spherical). The average agglomerate size in saline (Normosol, Nospira Inc., Lake Forest, IL) and 5% fetal bovine serum (FBS) was 191 nm as determined by dynamic light scattering via a Malvern Zetasizer version 7.01 (Westborough, MA). Finally, the valence state of the CeO_2_ NP was ~81% Ce^4+^ and ~19% Ce^3+^ determined by x-ray photoelectron spectroscopy (PHI 5000 Versaprobe XPS, Chanhassen, MN).

### Experimental animals

Male spontaneously hypertensive (SH) rats and Wistar-Kyoto (WKY) rats (~10 weeks old) were purchased from Harlan laboratories (Indianapolis, IN). SH rats were studied because they are a well-established model of microvascular dysfunction and excess local ROS production (Bakker et al., 2014; Brito et al., 2015). WKY rats were used as a normotensive control for the SH in these experiments (Okamoto et al., 1967). The rats were housed at the West Virginia University (WVU) Health Sciences Center Vivarium, in laminar flow cages, under controlled humidity and temperature, with a 12 h light/dark cycle, and food and water were provided *ad libitum*. The animals were acclimated for a minimum of 2 days prior to use. The WVU Institutional Animal Care and Use Committee at WVU approved all procedures.

### CeO_2_ NP solution preparation and exposure

The animals used in these studies were injected with a 100 μg dose (~0.42 mg/kg) of CeO_2_ NP 24 h prior to experimental assessments. To prepare this dose, a stock suspension was prepared daily containing 1.1 mg of dry CeO_2_ NP powder and 10 ml of Normosol with 5% FBS to reduce agglomeration (Minarchick et al., 2013, 2015). The CeO_2_ NP were vortexed for 5 min and then sonicated on ice for an additional 5 min. Rats were lightly anesthetized with isoflurane gas (5% induction, 2–3.5% maintenance). Rats were divided into four groups and were intravenously injected in the tail vein with a 900 μl bolus dose of saline (WKY-Sham, SH-Sham) or CeO_2_ NP stock suspension (WKY-CeO_2_ NP, SH-CeO_2_ NP) using a 23G needle. The final dose was 100 μg per rat. This dose was predetermined to cause a 50% impairment in microvascular function in previous experiments and was also within the functional dose range for CeO_2_ NP (Kim et al., 2012; Minarchick et al., 2015)Rats were monitored after treatment until they regained consciousness.

### Intravital microscopy preparation

Animals were anesthetized with Inactin [100 mg/kg, intraperitoneal (ip) injection] and placed on a heating pad to maintain a 37°C rectal temperature. The trachea was intubated to maintain a patent airway, and the right carotid artery was cannulated to monitor mean arterial pressure (MAP) and heart rate. A loop of the small intestine (ileum) was surgically exteriorized. Once exteriorized, two small incisions 6–10 cm apart were made along the intestinal wall using thermal cautery and the chyme was flushed from the lumen through the incisions. The ileum was then carefully placed over a clear pedestal, and gently secured so that the mesenteric circulation was at its *in situ* length. Throughout the preparation and experiment, the exposed mesentery was continuously superfused with an electrolyte solution (119 mM NaCl, 25 mM NaHCO_3_, 6 mM KCl, and 3.6 mM CaCl_2_) that was warmed to 37°C and equilibrated with 95% N and 2–5% CO_2_ (pH 7.35–7.40). Intestinal motility was suppressed using isoproterenol hydrochloride (10 mg/l, Sigma-Aldrich, St. Louis MO) and phenytoin (20 mg/l, Sigma-Aldrich, St. Louis MO). At these concentrations, isoproterenol and phenytoin have no significant effect on arteriolar tone (Bohlen et al., 1978). The superfusate rate was maintained at 4–6 ml/min to minimize equilibration with atmospheric oxygen (Boegehold and Bohlen, 1988). The animal preparation was then transferred to the stage of an intravital microscope coupled to a charge-coupled device (CCD) video camera. Observations were made with a 20x water immersion objective. Video images were displayed on a high-resolution color video monitor and recorded for off-line analysis. During analysis, arteriolar inner diameters were measured with a video caliper and one to three arterioles were studied per rat.

### Microvascular analysis

Arteriolar endothelium-dependent dilation was evaluated using acetylcholine (ACh, 0.25 M). ACh was iontophoretically applied to individual 5th order arterioles (20, 100, and 150 nA). Glass micropipettes were beveled at a 23–25° angle with 2–4 μm inner diameter tip and filled with ACh (Nurkiewicz et al., 2004). The pipette tip was placed in light contact with the arteriolar wall and a current programmer (Model 260; World Precision Instruments, New Haven, CT) was used to deliver continuous ejection currents (2 min). A recovery period (at least 2 min) followed each application. The ACh response determination was repeated in the presence of several chemical interventions which were applied via syringe pump (0.4 ml/min) into the superfusate delivery line for 30 min. The mesenteric loop was incubated with N^G^-monomethyl-L-arginine (L-NMMA, 10^−4^ M), a NOS inhibitor, to assess the influence of NO. Incubation with indomethacin (INDO, 10^−5^ M), a cyclooxygenase (COX) inhibitor, was used to assess the influence of COX products. The mesentery was also incubated with 2,2,6,6-tetramethylpiperidine-N-oxyl (TEMPOL, 10^−4^ M), a superoxide dismutase mimetic, and catalase (50 units/ml), a hydrogen peroxide scavenger, to assess the influence of ROS. All four chemical interventions (L-NMMA, INDO, TEMPOL, and catalase) were applied to the mesentery to determine influence of NO, COX products, and ROS simultaneously. Vascular smooth muscle responsiveness to NO was assessed using sodium nitroprusside (SNP, 0.05 M). SNP was iontophoretically applied to arterioles as previously described (10, 50, and 150 nA) (Nurkiewicz et al., 2004). Microvascular reactivity assessments and ejection currents were randomized and two to three assessments were completed per rat. At the end of each experiment, adenosine (ADO, 10^−4^ M) was added to the superfusate via syringe pump to determine the passive diameter of each arteriole studied.

Finally, leukocyte flux was assessed in mesenteric paired venules to quantify potential microvascular inflammation. Leukocytes that were either stationary or moving but maintained consistent contact with the venular wall for at least 200 μm were counted for 1 min in each venule studied.

### Oxidative stress analysis

A separate subset of animals (~10 weeks old) was used to determine changes in microvascular oxidative stress via fluorescent analysis. SH and WKY rats were exposed to either saline or CeO_2_ NP as previously described. Twenty-four hour post-exposure, the animals were anesthetized with Inactin (100 mg/kg, ip injection) and were placed on a heating pad to maintain a 37°C rectal temperature. The right carotid artery was cannulated to acquire blood for subsequent cytokine analysis (see Section Pro-inflammatory Cytokine Analysis). The mesentery was removed and placed in a dissecting dish with physiological salt solution (PSS) maintained at 4°C (Minarchick et al., 2015). Mesenteric arterioles (4th and 5th order) were isolated, transferred to a vessel chamber, cannulated between two glass pipettes, and tied with silk sutures (Living Systems Instrumentation, Burlington, VT). In the absence of light, dihydroethidium (DHE, 10^−4^ M) was intraluminally infused for 20 min followed by a 20 min wash with PSS to remove excess DHE. Prior to imaging, the arterioles were pressurized to 80 mm Hg using a servo control system (Sun et al., 1992). The vessel chamber was placed on an Olympus BX51WI upright microscope for imaging. The arteriole was first imaged briefly under traditional bright field settings. The arteriole was illuminated with a mercury lamp for 1 s with the appropriate excitation and emission filters for detection of ethidium bromide fluorescence (480–550 nm bandpass, 590 nm barrier) and hydroethidine fluorescence (330–385 nm bandpass, 590 nm barrier). DHE was used because it easily permeates cell membranes and can be oxidized by ROS to form ethidium bromide which intercalates into nuclear DNA (Morgan et al., 1979; Benov et al., 1998).

ROS-associated fluorescence was quantified in the arteriolar wall. A user-defined region of interest (ROI) box (10 × 100 μm) was placed vertically on the endothelial cell layer of the bright field image. It was previously determined that the mesenteric arteriolar wall thickness was ~13 μm; therefore, the ROI box encompassed the entire endothelial cell layer and most of the vascular smooth muscle layer. The images were analyzed using open access imaging software (ImageJ). The hydroethidine image represented background and non-specific fluorescence, therefore in order to analyze only fluorescence which had been intercalated into the DNA this fluorescence was removed from the ethidium bromide image. The ROI was superimposed on the resulting image in order to acquire the mean fluorescence of the arteriole. Total fluorescence intensity was calculated as average fluorescence intensity per pixel x surface area.

### Pro-inflammatory cytokine analysis

The blood collected from the SH and WKY rats was centrifuged (1100 × g) to separate the blood components. The plasma was collected, flash frozen in liquid nitrogen, and stored in a −80°C freezer until analysis. A pro-inflammatory cytokine multi-spot assay was completed per manufacturer's directions (MesoScale Diagnostics, Rockville, MD). The pro-inflammatory cytokines assessed were: interferon gamma (IFN-γ), interleukins-1 beta, 4, 5, 6, 10 and 13 (IL-1β, IL-4, IL-5, IL-6, IL-10, and IL-13), tumor necrosis factor alpha (TNF-α) and keratinocyte chemoattractant/human growth-regulated oncogene (KC/GRO).

Equations: Basal tone was calculated by the following equation,

$$\text{Basal Tone }(\%)=[({\text{D}}_{\text{M}}-{\text{D}}_{\text{BD}})/{\text{D}}_{\text{M}}]\times 100,$$

where D_BD_ is the baseline diameter measured prior to any current application, and D_M_ is the passive diameter recorded in the presence of ADO. The experimental responses to ACh and SNP are expressed using the following equation:

$$\begin{array}{l} \text{Diameter }(\text{Percent Maximal Response})=\\ \;[{\text{D}}_{\text{SS}}-{\text{D}}_{\text{BD}})/({\text{D}}_{\text{M}}-{\text{D}}_{\text{BD}})]\times 100, \end{array}$$

where D_SS_ is the maximal diameter recorded at each current.

### Statistics

Data are expressed as means ± standard error (SE). Point-to-point differences in the dose-response determinations were evaluated using Two-Way repeated measures analysis of variance (ANOVA) with a Student-Newman-Keuls *post-hoc* analysis when significance was found. Statistical differences in the slopes of the dose-response determinations were determined by linear regression analysis. The animal characteristics, arteriole characteristics, oxidative stress, and pro-inflammatory cytokine results were analyzed using an One-Way ANOVA with a Student-Newman-Keuls *post-hoc* analysis when significance was found. All statistical analysis was completed with SigmaPlot 11.0 (San Jose, CA) and GraphPad Prism 5 (San Diego, CA). Significance was set at *p* ≤ 0.05, n is the number of arterioles, and N is the number of animals.

## Results

### Animal and arteriolar characteristics

There were no significant changes in age or heart rate between the four groups (Table 1). MAP and heart weight were significantly increased in the SH-Sham and SH-CeO_2_ NP groups compared to the normotensive WKY groups (Table 1). These findings are consistent with SH rat characteristics. There was also a significant increase in body weight in the SH-CeO_2_ NP group compared to the WKY groups (Table 1). Finally, there were no observed differences in starting arteriolar diameter, maximal diameter, or basal tone between the four groups (Table 2). These results indicate that CeO_2_ NP injection exposure does not influence blood pressure or arteriolar tone, when comparing the WKY and SH groups.

**Table 1:** **Animal characteristics**.

**Group**	***N***	**Age (wks)**	**Weight (g)**	**Heart weight (g)**	**MAP (mm Hg)**	**Heart rate (beats/min)**
WKY-Sham	22	10.48±0.30	230.5±5.99	0.97±0.03	66.25±2.33	446.20±25.03
WKY-CeO_2_ NP	19	10.42±0.18	226.7±3.73	0.93±0.02	69.29±2.23	471.24±7.21
SH-Sham	24	10.08±0.32	245.7±6.20	1.12±0.03^*^^†^	113.33±5.37^*^^†^	427.83±18.49
SH-CeO_2_ NP	24	10.17±0.33	253.0±5.41^*^^†^	1.14±0.04^*^^†^	106.66±5.27^*^^†^	494.37±12.69

*Values are means ± SE; N, number of animals; MAP, mean arterial pressure*.

* p ≤ 0.05 vs. WKY-Sham;

† *p ≤ 0.05 vs. WKY-CeO_2_ NP*.

**Table 2:** **Arteriole characteristics**.

**Group**	***N***	**Starting diameter (μm)**	**Maximal diameter (μm)**	**Basal tone (%)**
WKY-Sham	42	59.35 ± 2.67	88.00 ± 3.40	32.75 ± 1.25
WKY-CeO_2_ NP	40	59.04 ± 2.65	92.60 ± 3.65	35.42 ± 1.53
SH-Sham	58	57.44 ± 2.30	85.81 ± 3.18	32.82 ± 1.16
SH-CeO_2_ NP	50	60.21 ± 2.65	96.80 ± 4.08	37.02 ± 1.49

*Values are means ± SE; n, number of arterioles*.

### Endothelium-dependent dilation

**Figure 1:**
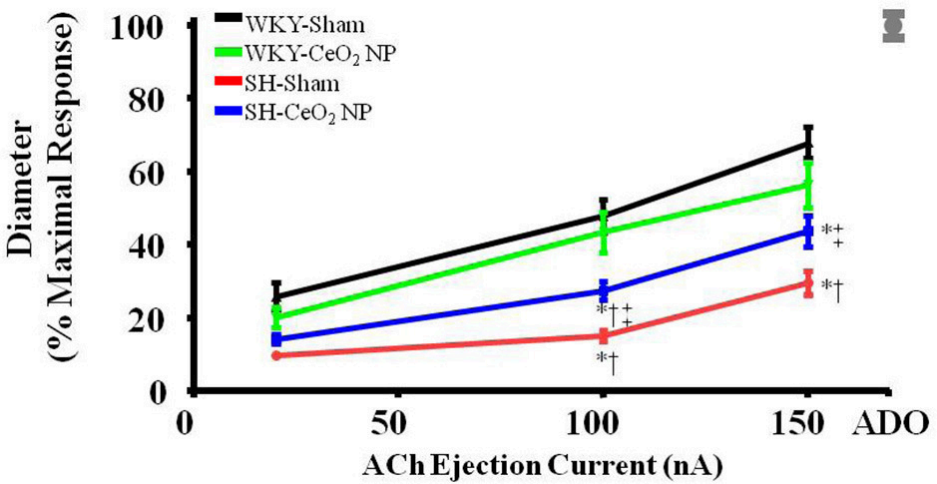
**ACh-induced vasodilation was impaired in arterioles from SH-Sham animals but was significantly improved in the SH-CeO_2_ NP group compared to the SH-Sham (*n* = 12–29)**. The gray mark represents the maximal dilation observed during incubation with ADO at the end of the experiment. ^*^p ≤ 0.05 vs. WKY-Sham, ^†^*p* ≤ 0.05 vs. WKY-CeO_2_ NP, ^‡^*p* ≤ 0.05 vs. SH-Sham.

Endothelium-dependent dilation was significantly impaired in the SH-Sham group (29.68 ± 3.28%, maximal response) compared to the WKY groups (53.92 ± 4.22%) (Figure 1). Following the 100 μg injection of CeO_2_ NP, there was a significant augmentation in endothelium-dependent dilation in the SH-CeO_2_ NP (43.76 ± 4.33%) compared to the SH-Sham group (29.68 ± 3.28%) (Figure 1). However, this dilation was still significantly impaired compared to the WKY-Sham group (53.92 ± 4.22%) (Figure 1). This data provides evidence that the injected CeO_2_ NP partially improve the microvascular dysfunction associated with hypertension.

### Endothelium-independent dilation

**Figure 2:**
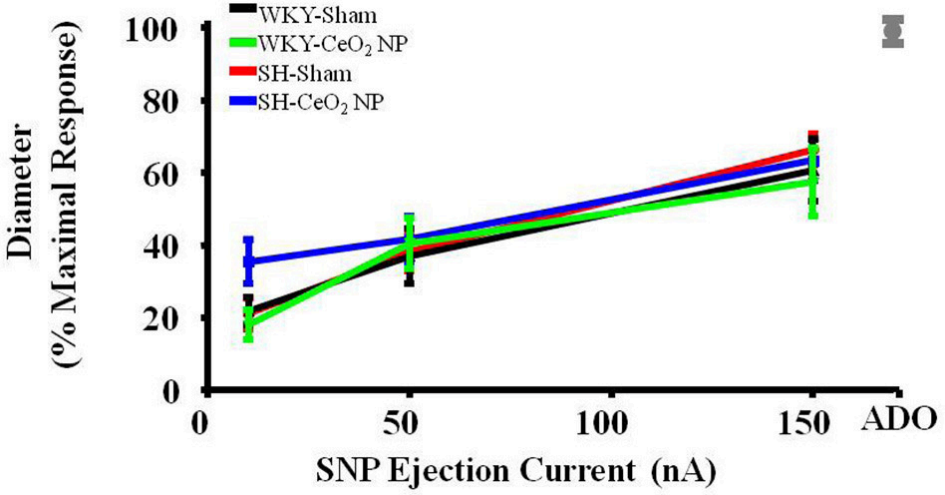
**Vascular smooth muscle responsiveness to NO was not impaired in the Sham groups or following CeO_2_ NP exposure (WKY-CeO_2_ NP and SH-CeO_2_ NP groups; *n* = 9–19)**. The gray mark represents the maximal dilation observed during incubation with ADO at the end of the experiment.

Endothelium-independent dilation was not impaired in the SH or WKY groups (Figure 2). This indicates that CeO_2_ NP injection exposure and hypertension, either alone or together, do not impair arteriolar vascular NO smooth muscle sensitivity.

### Contribution of NO, COX products, and ROS to ACh-induced vasodilation

**Figure 3:**
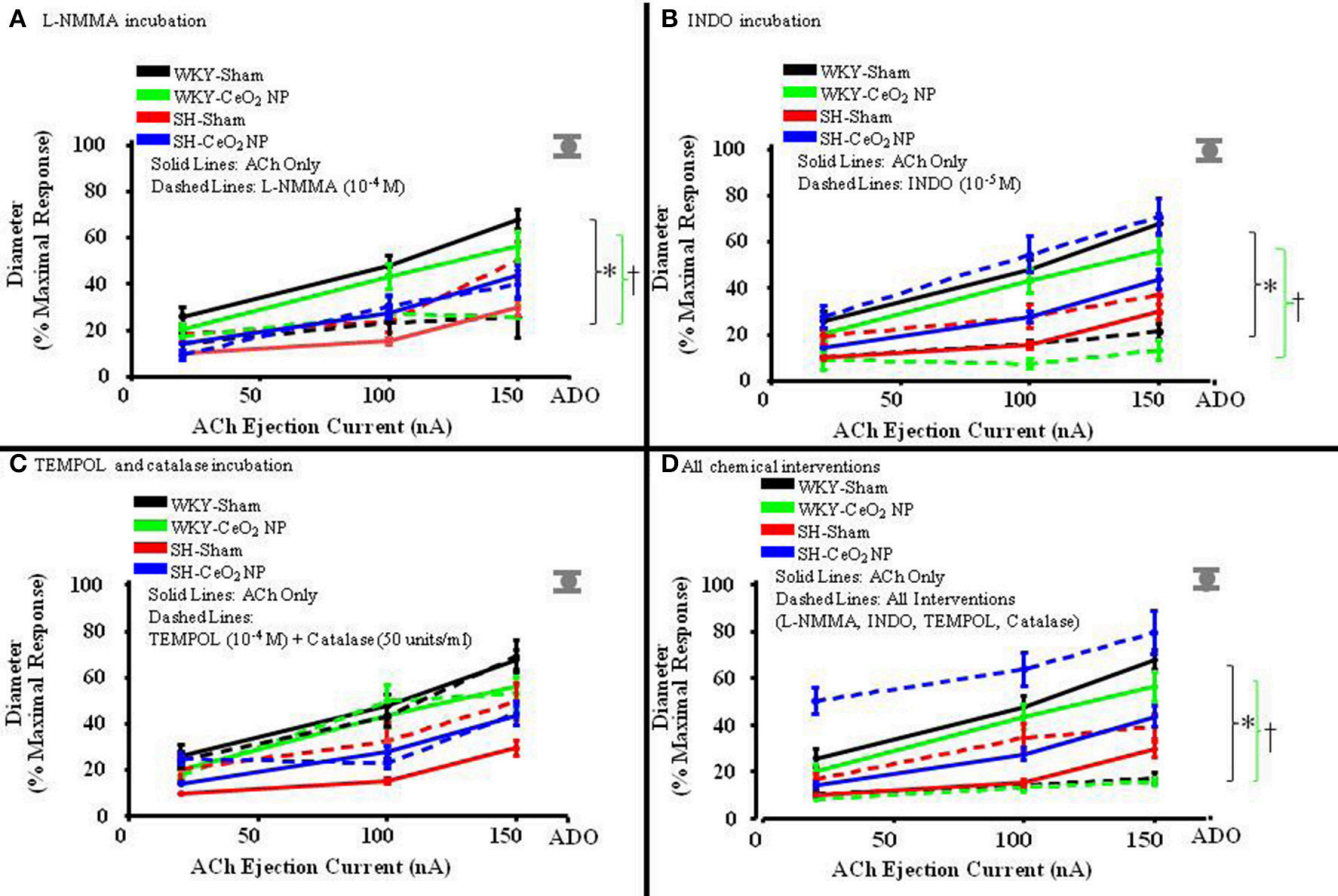
**The line graphs are the arterioles overall response to the various chemical interventions. (A)** The role of NO was assessed during incubation with L-NMMA (*n* = 14–18). This incubation resulted in a significant difference in the overall slopes of the dose-response determination within the WKY groups only. **(B)** The role of COX products was assessed during incubation with INDO (*n* = 9–14). This incubation resulted in a significant difference in the overall slopes of the dose-response determination within the WKY groups only. **(C)** Local ROS was scavenged during incubation with TEMPOL and catalase (*n* = 6–18). This incubation resulted in no changes in the overall slopes of the dose-response determination in any of the four treatment groups. **(D)** ACh-induced vasodilation was assessed during co-incubation with the four chemical interventions (*n* = 10–15). This incubation resulted in a significant difference in the overall slopes of the dose-response determination within the WKY groups only. The gray mark represents the maximal dilation observed during incubation with ADO at the end of the experiment. The brackets indicated significant differences in the overall slopes of the dose-determination. ^*^p ≤ 0.05 vs. WKY-Sham, ^†^*p* ≤ 0.05 vs. WKY-CeO_2_ NP.

**Figure 4:**
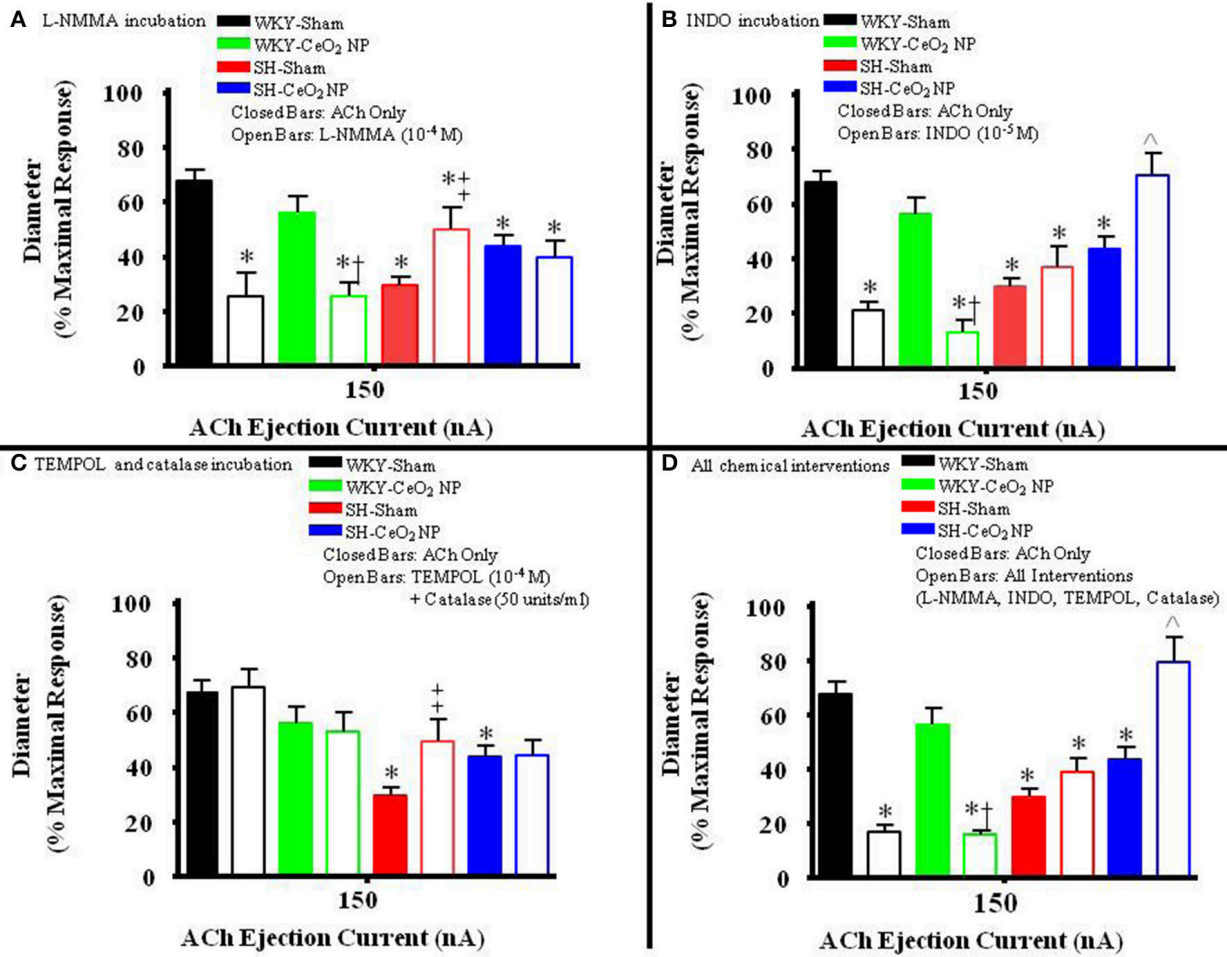
**The bar graph is the dilation that was observed at 150 nA for each animal group in the presence of the selected chemical intervention. (A)** The role of NO was assessed during incubation with L-NMMA (*n* = 14–18). This incubation resulted in a significant decrease in arteriolar vasodilation in the WKY groups. There was a significant increase in vasodilation during incubation in the SH-Sham group but there was no change in ACh-induced dilation in the SH-CeO_2_ NP group. **(B)** The role of COX products was assessed during incubation with INDO (*n* = 9–14). This incubation resulted in a significant decrease in arteriolar vasodilation in the WKY groups. There was no change in vasodilation in the SH-Sham group, but there was a significant increase in vasodilation following CeO_2_ NP (SH-CeO_2_ NP). **(C)** Local ROS was scavenged during incubation with TEMPOL and catalase (*n* = 6–18). This incubation resulted in no change in arteriolar vasodilation in the WKY groups and the SH-CeO_2_ NP group. There was increase in vasodilation in the SH-Sham group during incubation. **(D)** ACh-induced vasodilation was assessed during co-incubation with the four chemical interventions (*n* = 10–15). This incubation resulted in a significant decrease in arteriolar vasodilation in the WKY groups. There was no change in vasodilation in the SH-Sham group but there was a significant increase in vasodilation following CeO_2_ NP (SH-CeO_2_ NP). The open bars represent responses to ACh in the presence of a chemical mediator. ^*^p ≤ 0.05 vs. WKY-Sham, ^†^*p* ≤ 0.05 vs. WKY-CeO_2_ NP, ^‡^*p* ≤ 0.05 vs. SH-Sham, ^∧^*p* ≤ 0.05 vs. SH-CeO_2_ NP.

The role of NO in ACh-induced vasodilation was assessed during incubation with L-NMMA. Incubation of the mesentery with L-NMMA significantly reduced vasodilation in response to ACh, both in terms of the overall slopes and at the highest ejection current, in the WKY-Sham and WKY-CeO_2_ NP groups (53.92 ± 4.22%, and 56 ± 6.13%, respectively vs. 11.86 ± 9.89%, and 25.81 ± 5.08% during incubation) (Figures 3A, 4A). Additionally, there was a significant improvement in dilation in the SH-Sham during incubation with L-NMMA (29.68 ± 3.28% vs. 50.18 ± 8.09% during incubation), but there was no improvement in arteriolar dilation during incubation in the SH-CeO_2_ NP group (Figure 4A).

The role of COX products were assessed during incubation with INDO. There was also a significant impairment in arteriolar reactivity, in regards to the overall slopes and at the highest ejection current, during incubation with INDO in the WKY-Sham and WKY-CeO_2_ NP groups (53.92 ± 4.22%, and 56 ± 6.13%, respectively vs. 27.06 ± 4.84%, and 13.12 ± 4.28% during incubation) (Figures 3B, 4B). There was no change in vasodilation during INDO incubation in the SH-Sham group (Figure 4B). Finally, there was a significant increase in vasodilation in the SH-CeO_2_ NP during INDO incubation (43.76 ± 4.33% vs. 70.73 ± 7.92% during incubation) (Figure 4B).

Local ROS was scavenged during simultaneous incubation with TEMPOL and catalase. There was no change in arteriolar vasodilation during TEMPOL and catalase incubation in the WKY-Sham, WKY-CeO_2_ NP, and SH-CeO_2_ NP groups (Figures 3C, 4C). There was a significant increase in the response to ACh in the SH-Sham group during incubation with these exogenous anti-oxidants (29.68 ± 3.28% vs. 49.62 ± 7.86% during incubation) (Figure 4C).

Finally, arteriolar dilation was assessed during co-incubation with all four chemical interventions. In regards to the overall slopes and the highest ejection current, there was significant decrease in arteriolar reactivity in the WKY-Sham and WKY-CeO_2_ NP 53.92 ± 4.22%, and 56 ± 6.13%, respectively vs. 7.08 ± 11.26%, and 15.82 ± 1.50% during incubation) (Figures 3D, 4D). In the SH groups, there was no significant changes in arteriolar vasodilation in the SH-Sham group, but there was a significant increase in dilation in the SH-CeO_2_ NP group (43.76 ± 4.33% vs. 72.33 ± 6.26% during incubation) (Figure 4D). Overall, these data provide initial evidence that there are differential contributions of NO, COX products, and ROS to the observed vasodilation after CeO_2_ NP injection exposure in the SH rats. The increase in microvascular reactivity during TEMPOL and catalase incubation in the SH-Sham group (which was similar to the SH-CeO_2_ NP group), provides evidence that CeO_2_ NP may act as an anti-oxidant *in vivo*.

### Microvascular oxidative stress

**Figure 5:**
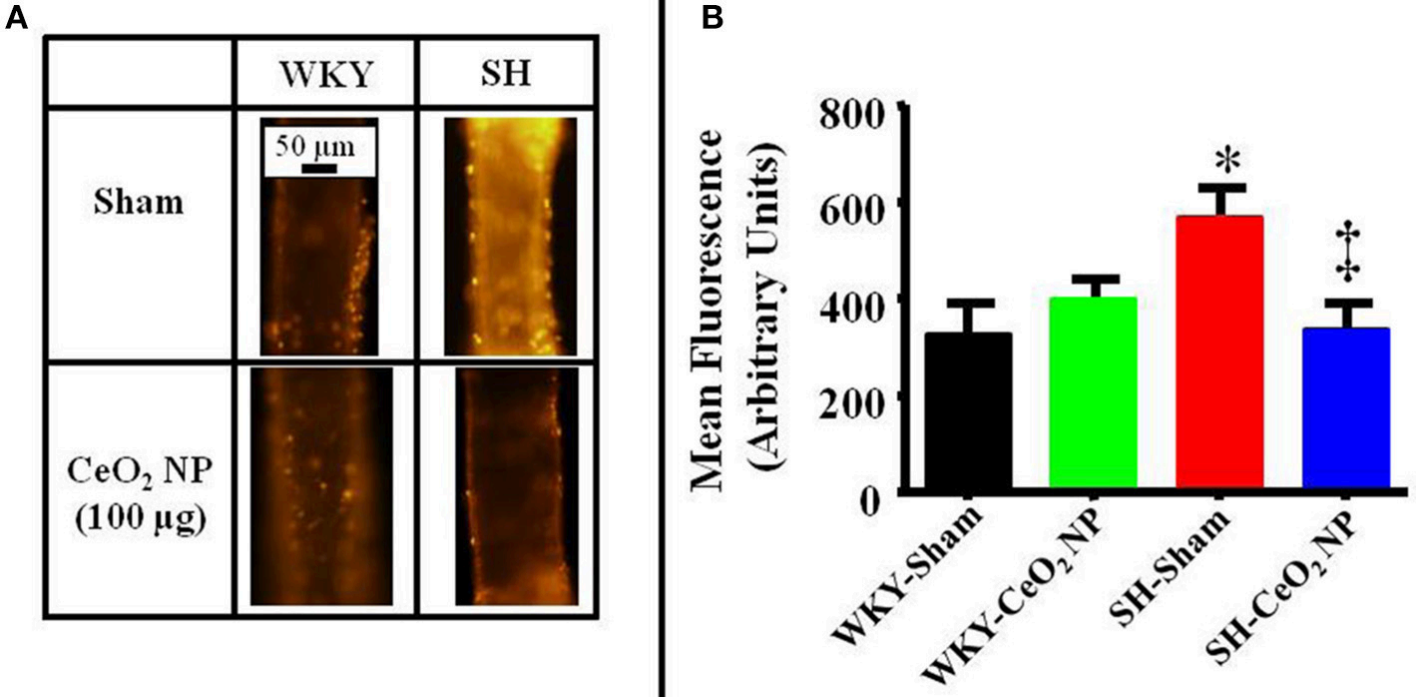
**(A)** Representative ethidium bromide images indicating an increase in oxidative stress in the SH-Sham group. **(B)** Quantitative analysis of the ethidium bromide images revealed a significant increase in vascular oxidative stress in the SH-Sham group that was reduced in the SH-CeO_2_ group (*n* = 5–7). These levels were similar to the WKY-Sham and WKY-CeO_2_ NP groups. ^*^p ≤ 0.05 vs. WKY-Sham, ^‡^*p* ≤ 0.05 vs. SH-Sham.

There was a significant increase in microvascular oxidative stress in the SH-Sham group compared to the WKY groups (Figures 5A,B). The level of oxidative stress in the SH rats was significantly decreased following CeO_2_ NP exposure and the level of oxidative stress in the SH-CeO_2_ NP group was similar to the WKY-Sham group (Figures 5A,B). There were also no significant differences between the WKY-Sham and WKY-CeO_2_ NP groups (Figures 5A,B). Taken together, these results provide evidence that CeO_2_ NP have anti-oxidant activity *in vivo*.

### Systemic inflammation and biomarkers

**Figure 6:**
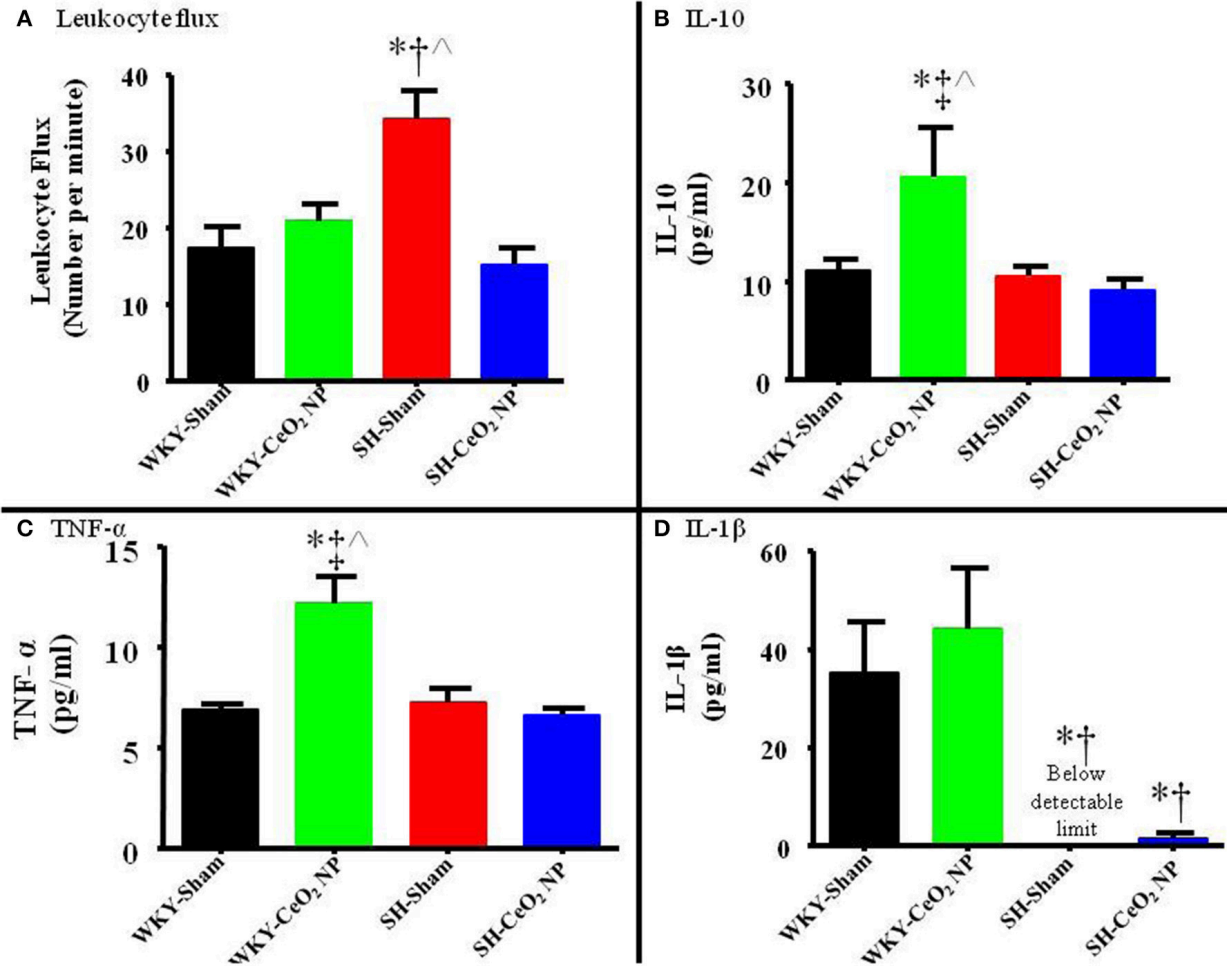
**(A)** Leukocyte flux was assessed in venules during intravital microscopy. There was a significant increase in leukocyte flux in the SH-Sham group, which was decreased following CeO_2_ NP exposure (*n* = 12–15). **(B)** Plasma IL-10 levels were significantly increased in the WKY-CeO_2_ NP, but not in the other three groups (*N* = 7). **(C)** Plasma TNF-α levels were significantly increased in the WKY-CeO_2_ NP, but not in the other three groups (*N* = 6–7). **(D)** Plasma IL-1β levels were significantly decreased in the SH-Sham and SH-CeO_2_ NP groups, but were unaltered in the WKY-CeO_2_ NP group (*N* = 6–7). ^*^p ≤ 0.05 vs. WKY-Sham, ^†^*p* ≤ 0.05 vs. WKY-CeO_2_ NP, ^‡^*p* ≤ 0.05 vs. SH-Sham, ^∧^*p* ≤ 0.05 vs. SH-CeO_2_ NP.

Venular leukocyte flux was assessed in all four groups. There was a significant increase in leukocyte flux in the SH-Sham group compared to the WKY-Sham (34 ± 4 vs. 17 ± 3 cells/min), and WKY-CeO_2_ NP (34 ± 4 vs. 21 ± 2 cells/min) groups (Figure 6A). This flux was significantly decreased in the SH animals following exposure to CeO_2_ NP (15 ± 2 vs. 34 ± 4 cells/min) (Figure 6A). There was also no change in leukocyte flux between the WKY-Sham and WKY-CeO_2_ NP groups (Figure 6A). Pro-inflammatory cytokines were analyzed in harvested plasma. There were no significant differences in IFN-γ, IL-4, IL-5, IL-6, IL-13, and KC/GRO between the groups (data not shown). There was a significant increase in IL-10 and TNF-α in the WKY-CeO_2_ NP group compared to the other three groups (IL-10: 20.60 ± 4.92 pg/ml vs. 10.24 ± 1.09 pg/ml, average of WKY-Sham, SH-Sham, and SH-CeO_2_ NP groups; TNF-α: 12.12 ± 1.38 pg/ml vs. 6.9 ± 0.45 pg/ml, average of WKY-Sham, SH-Sham, and SH-CeO_2_ NP groups) (Figures 6B,C). Finally, there was a decrease in IL-1β in the SH-Sham and SH-CeO_2_ NP groups compared to both WKY groups (1.58 ± 1.03 pg/ml vs. 39.64 ± 11.37 pg/ml, average of WKY-Sham and WKY-CeO_2_ NP groups) (Figure 6D). Taken together these results provide evidence that injected CeO_2_ NP may alter inflammatory signaling pathways in both normotensive and hypertensive animals.

## Discussion

To our knowledge, this is the first study to investigate the *in vivo* anti-oxidant potential of CeO_2_ NP in a hypertensive animal model. The first major finding of this study is that CeO_2_ NP have anti-oxidant potential *in vivo* resulting in decreased microvascular dysfunction and oxidative stress that is associated with hypertension. The second finding of this study is that there were changes in leukocyte adhesion/rolling and cytokine expression, which indicated that CeO_2_ NP might influence thrombosis risk and inflammation.

Intravenously injected CeO_2_ NP significantly reduced the level of oxidative stress in the SH rats, which contributed to decreased microvascular dysfunction. This finding supports the notion that CeO_2_ NP act as an anti-oxidant, and this finding is corroborated by numerous studies (Colon et al., 2010; Estevez et al., 2011; Kim et al., 2012). Despite this positive result, previous studies have shown a decrease in vascular function following CeO_2_ NP exposure (Wingard et al., 2011; Minarchick et al., 2015). These disparate effects may be due to changes in the basal level of ROS and the animal model.

SH rats have an increased basal level of ROS, that was not present in the Sprague-Dawley rats that were used in previous studies (LeBlanc et al., 2010). An increase in ROS generation is also observed during radiation treatments and stroke, where these ENM were shown to be beneficial in a therapeutic capacity. This conceptual need for an increased basal level of ROS is also supported *in vitro*. Cardiac progenitor cells and cardiomyocytes were protected from high ROS levels when CeO_2_ NP were present (Niu et al., 2011; Pagliari et al., 2012). Furthermore, there were not high initial levels of ROS production in studies that reported an increase in ROS following CeO_2_ NP exposure (Park et al., 2008; Horie et al., 2011). When taken together these studies support the hypothesis that CeO_2_ NP work optimally as an anti-oxidant when there are elevated levels of ROS prior to or in conjunction with exposure.

In addition to the potential changes in ROS, the selected animal model may also affect the lack of microvascular influences following CeO_2_ NP in the WKY rats. Exposures to ENM and particulate matter have yielded different results in various strains of animals, which indicated that genetic background may play a vital role in determining the *in vivo* effects of nanomaterials (Gottipolu et al., 2009; Wingard et al., 2011). Both the WKY and SH animals are inbred strains compared to the outbred Sprague-Dawley rat used in other studies with CeO_2_ NP. Genetic modifications have been shown to alter the effects of ENM exposure (Wingard et al., 2011). CeO_2_ NP exposed mice have an increase in pulmonary inflammation and vascular dysfunction; however, when these mice were genetically modified to knockout mast cell production the inflammatory and vascular effects were absent (Wingard et al., 2011). Therefore, it is reasonable to speculate that genetic differences between the WKY, SH, and Sprague-Dawley rat lineages may contribute to the differences in the observed microvascular reactivity. Further studies are required to investigate potential genetic differences and their influence on the cardiovascular outcomes following ENM exposure.

Finally, the experimental techniques utilized in various vascular function assessments may also have contributed to the differential microvascular effects observed in this and other studies (Wingard et al., 2011; Minarchick et al., 2015). Previous microvascular assessments were completed using pressure or wire myography. These techniques allowed for the determination of specific influences of certain agonists without the confounding influences of blood flow and autonomic innervation (Minarchick et al., 2015). However, the vascular assessments in this manuscript were conducted using intravital microscopy to determine if the observations from previous experiments persisted in the whole animal. Therefore, the lack of overt endothelium-dependent and -independent microvascular dysfunction in the WKY rats could be due in part to the activation of compensatory mechanisms, sympathetic stimulation, and/or blood flow, which may hide underlying microvascular dysfunction.

Despite the partial improvement in microvascular dysfunction after exposure, CeO_2_ NP could not completely restore normal microvascular function in the SH animal model (Figure 1). This lack of an overall improvement could be due to arachidonic acid (AA) metabolism via COX. This study provides evidence that CeO_2_ NP exposure results in an apparent shift between NOS and COX activity, resulting potentially in increased prostaglandin production. Furthermore, the changes in AA signaling appear to be unique to the CeO_2_ NP exposure in the SH animal model. An increase in prostaglandin production can affect microvascular reactivity by influencing both vasodilation and vasoconstriction. Based on improved vasodilation during INDO incubation in the SH-CeO_2_ NP animals, it can be speculated that CeO_2_ NP exposure increases thromboxane A_2_ activity, which would result in vasoconstriction (Figures 3, 4). This increased influence on vasoconstriction could prevent the arterioles from fully dilating in response to ACh. In addition to potential thromboxane A_2_ alterations, a potential increase in arteriolar vasoconstriction could be due to changes in autonomic regulation, which maybe from changes in sympathetic stimulation. It should also be noted there was an increase in dilation during incubation with L-NMMA in the SH-Sham animals. The source of this improved dilation is unknown, but may be linked to change in signaling in the hypertensive model, including NOS uncoupling, increased prostacyclin production and improved endothelium-derived hyperpolarizing factor signaling (Nishikawa et al., 2000; Parkington et al., 2008). Additional studies will be needed to determine the influence of CeO_2_ NP on AA signaling, COX activation, and autonomic regulation.

This study provides promising evidence of the *in vivo* anti-oxidant potential of CeO_2_ NP; however, it is currently unclear if CeO_2_ NP are operating at their maximal efficiency *in vivo*. One of the primary factors that could affect the cardiovascular outcomes of CeO_2_ NP (and ENM in general) is the dose. The dose used in this study was ~0.42 mg/kg based on an average animal weight of 240 g. This dose was on the lower end of the published effective doses (0.4–0.7 mg/kg) used to protect mice from stroke damage (Kim et al., 2012). Therefore, it is reasonable to speculate that increasing the dose may improve CeO_2_ NP anti-oxidant effectiveness in hypertensive animals. Dose refinement could potentially result in a further improvement in microvascular function and decreased oxidative stress. This need for further dose refinement also highlights a limitation of this study. It was not possible to determine the amount of CeO_2_ NP that accumulated within the vascular wall; therefore, it is unclear at this time if the observed results are due to direct or indirect interactions with CeO_2_ NP. Independent of direct or indirect effects, understanding the cardiovascular influences, particularly in regards to the effective dose, is vital if CeO_2_ NP are to be developed as a pharmaceutical agent. Additionally, the effective dose needs to be better understood because research has shown that higher concentrations of CeO_2_ NP may result in decreased anti-oxidant activity and an increase in pro-oxidant activity (Horie et al., 2011). This shift in activity highlights the need to investigate various doses within a model, and additional dosing paradigms will need to be tested to explore the full potential of CeO_2_ NP to improve the microvascular dysfunction associated with hypertension.

Another factor that may influence the efficiency of CeO_2_ NP anti-oxidant activity is the primary size of the nanoparticles. The ENM used in this study and in another stroke study had a primary size of 2–4 nm; however, studies that used larger ENM (>20 nm) have been associated with an increase in ROS generation as opposed to a decrease (Park et al., 2008; Kim et al., 2012). This difference in the cellular effects has also been observed with other ENM. Silver nanoparticles with a diameter < 40 nm had increased cell permeability and toxicity vs. their larger counterparts (>40 nm) (Sheikpranbabu et al., 2010; Trickler et al., 2010). This not only illustrates the importance of characterizing the size of the ENM used, but also how a small change in ENM size can alter both their physiochemical characteristics and their cellular and cardiovascular effects.

The proposed anti-oxidant activity is most likely connected to the valence states (Ce^3+^ and Ce^4+^) that are present in CeO_2_ NP. Both valence states may react with any free radical, but research has shown that depending on the valence state this ENM will preferentially react with various free radicals. Under *in vitro* conditions, Ce^3+^ preferentially reacts with the superoxide and Ce^4+^ reacts with NO (Xu and Qu, 2014). The CeO_2_ NP used in this study were ~19% Ce^3+^ and 81% Ce^4+^ indicating they may react more readily with NO; however in this study CeO_2_ NP exposure did not appear to impair NO bioavailability. This apparent lack of NO scavenging may be due to changes in the valence state of CeO_2_ NP following exposure; however, it is currently unknown to what extent the valence state of CeO_2_ NP is modified following *in vivo* exposure (Hayes et al., 2002).

CeO_2_ NP exposure has also been associated with changes in inflammation. Similar to this study, others have shown an increase in pro-inflammatory cytokines, changes in the leukocyte flux, and inflammatory NOS expression following *in vivo* CeO_2_ NP exposure, but these results are often inconsistent (Hirst et al., 2009; Cho et al., 2010; Hussain et al., 2012). In this study, an increase in cytokine expression (e.g., TNF-α and IL-10) was present in the normotensive CeO_2_ NP exposed animals and it has been well established that inflammation is associated with microvascular dysfunction (Nurkiewicz et al., 2006; LeBlanc et al., 2009). Furthermore, an increase in TNF-α and IL-10 is associated with the activation of macrophages via toll-like receptors, which further supports CeO_2_ NP potential to modify inflammation (Mosser, 2003). Finally, it should be noted that it is unclear if CeO_2_ NP preferentially effect ROS or inflammation and further studies are needed to address this issue.

Additionally, these changes in inflammatory cytokines and leukocyte flux could indicate that CeO_2_ NP modify thrombosis development. The increase in leukocyte flux in the SH rats provide indirect evidence for endothelial cell activation, which results in the production of various adhesion proteins and factors essential for fibrinolysis (e.g., plasminogen activator inhibitor-1). Plasminogen activator inhibitor-1 (PAI-1) is secreted by macrophages, endothelial cells and vascular smooth muscle cells. Furthermore, this factor is controlled by oxidative stress. Studies with nano-copper oxide have shown an increase in PAI-1 secretion following exposure and it is possible that this factor is also elevated in the SH-Sham rats, thus increasing the risk of thrombosis (Yu et al., 2010b). However, following CeO_2_ NP exposure there is a decrease in leukocyte flux and oxidative stress in the SH rats, which could indicate a decreased thrombosis risk. However, the potential increased thromboxane A_2_ activity (in the CeO_2_ NP exposed SH rats) may be linked to the activation of platelets, which would increase the risk of thrombosis in these animals. Overall, it appears that CeO_2_ NP influences the thrombotic potential in SH rats, but at this time, it is unclear if they increase or decrease this risk and further studies are needed to understand these influences.

It should also be noted that there was no microvascular impairment in the normotensive rats after exposure despite an increase in pro-inflammatory cytokines. This lack of impairment maybe due to the time post-exposure (24 h) when reactivity was assessed and the lack of increased leukocyte adhesion and rolling in the WKY group further supports this notion. If the animals were exposed for a longer period of time (over 24 h), an increase in the activation of adhesion molecules and, ultimately, leukocyte extravasation may lead to an increase in microvascular dysfunction and thrombosis development.

Finally, the time of inflammatory assessments may also contribute to the observed changes in circulating cytokines. TNF-α is classically considered a pro-inflammatory cytokine that is involved in macrophage activation, whereas IL-10 is often considered an anti-inflammatory cytokine because is it capable of inhibiting TNF-α and IFN-γ (Feghali and Wright, 1997). In this study, it is possible that at 24 h IL-10 is beginning to increase, whereas TNF-α may be beginning to decrease, and this change in expression could result in the brief elevation of both cytokines. Additionally, based on these results, it is reasonable to speculate that CeO_2_ NP may have anti-inflammatory potential due to the increase in IL-10. Furthermore, SH rats typically have chronic inflammation, which contributes to their microvascular dysfunction (Sanz-Rosa et al., 2005). This study showed limited changes in the expression of pro-inflammatory cytokines in the SH animals and this lack of cytokine production may due to the age of the animals used in this study. Additionally, these limited cytokine and leukocyte results highlight the need for an extensive time course study to fully understand the effects of CeO_2_ NP exposure on inflammation and thrombosis development. Further studies will need to investigate the relationship between CeO_2_ NP, the inflammatory response, and exposure time in normotensive and hypertensive rats.

In conclusion, this study provides evidence that injected CeO_2_ NP decreases microvascular oxidative stress in a high ROS environment, which in turn, improves microvascular function. These results further our understanding of CeO_2_ NP behavior *in vivo* and highlight their therapeutic potential as an anti-oxidant, particularly in pathologies associated with elevated ROS. Furthermore, this study showed changes in the inflammatory profile following CeO_2_ NP exposure. Similarly, it is reasonable to speculate that CeO_2_ NP used preemptively would oppose the acute escalation of local ROS levels associated with various injuries and treatments. These changes were primarily observed in the WKY group, which highlight the need for further research on this ENM in order to understand fully its influences on systemic inflammation. Fully understanding how CeO_2_ NP act *in vivo* in both low and high ROS environments is critical for the continued and expanded development of this potential therapeutic agent.

## Author contributions

VM performed the experiments in this manuscript. TN, PS, and VM all contributed to the development and experimental design of the experiments. Finally, ES provided the CeO_2_ NP and nanoparticle characterization needed for these studies. All authors reviewed and approved this manuscript prior to submission.

## Funding

This work was supported by the following sources: National Institutes of Health R01-ES015022 (TN), K99-ES024783 (PS), the National Science Foundation Cooperative Agreement-1003907 (TN), and DGE-1144676 (VM).

### Conflict of interest statement

The authors declare that the research was conducted in the absence of any commercial or financial relationships that could be construed as a potential conflict of interest.
